# Exercise Reverses the Alterations in Gut Microbiota Upon Cold Exposure and Promotes Cold-Induced Weight Loss

**DOI:** 10.3389/fphys.2020.00311

**Published:** 2020-05-04

**Authors:** Yan Meng, Lina Chen, Wentao Lin, Hongjuan Wang, Guoqin Xu, Xiquan Weng

**Affiliations:** ^1^College of Exercise and Health, Guangzhou Sport University, Guangzhou, China; ^2^Chang Jiang Yi Zhong, Jingde, China; ^3^Shenzhen Health Time Gene Technology Co., Ltd., Shenzhen, China

**Keywords:** microbiome, combined effect of cold and exercise, beige fat, cardiovascular risk, white fat browning proteins

## Abstract

Gut microbiota has been reported to contribute to reduced diet-induced obesity upon cold exposure. Furthermore, gut microbiome fermentation determines the efficacy of exercise for diabetes prevention and enhances exercise performance. However, there have been no systematic examinations of changes in gut microbiome composition in relation to exercise performed under low-temperature conditions. In this study, we investigated the effects of exercise performed under different conditions (room temperature, acute cold, intermittent cold, and sustained cold) in obese rats maintained on a high-fat diet at four time points during experimental trials (days 0, 1, 3, and 35), including observations on white fat browning, weight loss, cardiovascular effects, and changes in gut microbiota among treatment groups. We found that exercise under sustained cold conditions produced a remarkable shift in microbiota composition. Unexpectedly, exercise was found to reverse the alterations in gut microbiota alpha-diversity and the abundance of certain bacterial phyla observed in response to cold exposure (e.g., *Proteobacteria* decreased upon cold exposure but increased in response to exercise under cold conditions). Moreover, exercise under cold conditions (hereafter referred to “cold exercise”) promoted a considerably higher level of white fat browning and greater weight loss and protected against the negative cardiovascular effects of cold exposure. Correlation analysis revealed that cold exercise-related changes in gut microbial communities were significantly correlated with white fat browning and cardiovascular phenotypes. These results could reveal novel mechanisms whereby additional health benefits attributable to both cold and exercise are mediated *via* altered gut microbes differently compared with either of them alone.

## Introduction

Cold exposure can protect against excess weight gain and improve health, effects that are believed to be mediated in part by gut microbes. Cold exposure has been shown to markedly reduce populations of the intestinal bacterium *Akkermansia muciniphila* and alter the composition of other bacterial species in mice (Chevalier et al., 2015). Notably, this microbial shift has been demonstrated to be sufficient to burn brown fat, improve glucose metabolism, and reduce weight (Turnbaugh et al., 2006). Recent studies in mice have indicated that reduced ambient temperatures can promote alterations in the gut microbiota, which in turn enhance the thermogenic capacity of adipose tissues, and hence the energy expenditure of hosts (Chevalier et al., 2015; Ziętak et al., 2016). A further study has shown that exposure to cold promotes the conversion of cholesterol to bile acids and induces adaptive thermogenesis *via* the brown adipose tissue (BAT)–liver–gut axis (Worthmann et al., 2017).

Exercise is now known to promote proliferation in the populations of certain bacterial strains, including those of *Veillonella*, in elite athletes, and it is known that an altered microbiome can enhance performance in mice (Scheiman et al., 2019). Other studies have also provided evidence that exercise can promote changes in microbial composition and have described how these shifts could provide health-related benefits (Clarke et al., 2014; Lambert et al., 2015; Welly et al., 2016; Bressa et al., 2017; Monda et al., 2017; Allen et al., 2018; Cronin et al., 2018). Furthermore, a study using mice (Allen et al., 2017) and two human studies (Barton et al., 2017; Allen et al., 2018) have demonstrated that endurance exercise does have effects on some microbial species and microbiome function, such as the production of short-chain fatty acids that are beneficial for health, after adjusting for dietary habits.

Although both acute and prolonged cold exposure are known to induce hypertension and trigger cardiovascular complications (Ikäheimo, 2018), regular and endurance exercise has been shown to reduce cardiovascular risk factors (Munukka et al., 2018). Furthermore, short-term endurance exercise has been found to induce changes in the gut microbiota associated with cardiometabolic disease risk factors (Taniguchi et al., 2018). On the basis of these observations, it is reasonable to assume that an investigation of exercise interventions, particularly at low temperatures, could provide key insights into the associations among the gut microbiome, exercise, temperature, and systemic metabolism. In this study, using *16S rRNA* gene sequencing data, we sought to characterize the alpha-diversity (α-diversity) and structural dynamics of the gut microbiota in response to cold exposure alone, exercise alone, and a combination of cold exposure and exercise in obese mice maintained on a high-fat diet.

To the best of our knowledge, this study presents the first description of the combined effects of exercise and cold exposure on the gut microbiome. We show that gut microbiota α-diversity increases during cold or exercise but decreases upon exercise under cold conditions (hereafter referred to as “cold exercise”). The reverse alterations of gut microbiota occurring in response to both cold exposure and exercise suggested that the interactive effect between cold exposure and exercise on shaping the gut microbiota contributes to a greater weight loss and prevention of the negative cardiovascular effects attributable to cold exposure alone.

## Materials and Methods

### Animals

In this study, we performed a 12-week feeding intervention based on a high-fat, high-sugar diet to induce obesity in 40 4-week-old Sprague–Dawley rats that were obtained from Guangzhou Southern Medical University [ethical approval number: SCXK (Yue) 2011-0015]. The study was carried out in accordance with the principles of the Basel Declaration and recommendations of the Guangzhou Sport University protocol. The protocol was approved by the Guangzhou Sport University Ethics Committee (ethical approval number: 2017DWLL-001).

### Study Design and Dietary Intervention Protocol

The high-fat, high-sugar diet (protein, 19%; fat, 18.5%; carbohydrate, 50.5%) used in the present study was obtained from Guangdong Medical Laboratory Animal Center [SCXK (Guangdong) 2013-0002]. Adult rats at 16 weeks of age were randomly assigned to one of the following eight groups (five rats per group) for a 5-week intervention (Table 1): (i) control (Sport-N-Cold-0h), (ii) exercise alone (Sport-Y-Cold-0h), (iii) acute cold alone (Sport-N-Cold-4h), (iv) acute cold + exercise (Sport-Y-Cold-4h), (v) intermittent cold alone (Sport-N-Cold-28h), (vi) intermittent cold + exercise (Sport-Y-Cold-28h), (vii) sustained cold alone (Sport-N-Cold-168h), and (viii) sustained cold + exercise (Sport-Y-Cold-168h). The rats were maintained in individual cages under a 12-h light/12-h dark cycle, at 24°C–26°C (room temperature treatment) and 3°C–4°C (cold exposure treatment). The diet was recorded per group with mean ± standard deviation of 23.436 ± 4.109 g. Animal treadmill exercise was performed at a speed of 25 m/min and slope of 0°. On alternate days, the rats performed two 30-min bouts of exercise. On the final day of the intervention (day 35), samples of blood, inguinal fat cells, and brown fat cells were collected. We performed brown fat imaging from the shoulder blades and collected 160 brown fat samples for subsequent AMP-activated protein kinase (AMPK), peroxisome proliferator-activated receptor-gamma coactivator-1α (PGC-1α), and uncoupling protein 1 (UCP1) protein expression analyses. Collection of fecal samples and weight measurements were performed at baseline (day 0), during the intervention (days 1 and 3), and at the end of the intervention (day 35).

**TABLE 1:** Cold exercise experimental design.

Group	5-week cold time (h)	Exercise	Annotation	Intervention
Sport-N-Cold-0h	0	N	Control (NC)	24°C–26°C room temperature; no exercise
Sport-Y-Cold-0h	0	Y	Exercise alone (NE)	24°C–26°C room temperature; treadmill exercise for 1 h on alternate days
Sport-N-Cold-4h	4	N	Acute cold alone (AC)	24°C–26°C room temperature; no exercise; cold exposure (3°C–4°C) 4 h before sampling
Sport-Y-Cold-4h	4	Y	Acute cold + exercise (AE)	24°C–26°C room temperature; treadmill exercise for 1 h on alternate days; cold exposure (3°C–4°C) 4 h before sampling
Sport-N-Cold-28h	28	N	Intermittent cold alone (IC)	Daily cold exposure for 4 h, with the remaining time spent at room temperature. No exercise
Sport-Y-Cold-28h	28	Y	Intermittent cold + exercise (IE)	Daily cold exposure 4 h, with the remaining time spent at room temperature. Treadmill exercise for 1 h on alternate days
Sport-N-Cold-168h	168	N	Sustained cold alone (SC)	Cold exposure (3°C–4°C); no exercise
Sport-Y-Cold-168h	168	Y	Sustained cold + exercise (SE)	Cold exposure (3°C–4°C); treadmill exercise for 1 h on alternate days

### Phenotyping

Fat and lean masses were quantified by nuclear magnetic resonance (Bruker), with the energy contents of fat and lean masses being expressed in kilojoules. At the end of the experiment, we performed glucose and insulin tolerance tests, and Western blot analysis was undertaken to determine the abundance of AMPK, PGC-1α, and UCP1 proteins.

### Isolation of Bacterial Genomic DNA

Bacterial genomic DNA was extracted from stool samples using an E.Z.N.A. Stool DNA Kit (Omega Bio-tek, Norcross, GA, United States) according to the manufacturer’s protocol, and a GeneMATRIX Stool DNA Purification Kit (EURx) was used to purify the extracted DNA, which was isolated using the repeated bead beating method (Rühlemann et al., 2016).

The V3–V4 region of the bacterial 16S ribosomal RNA gene was amplified by PCR (95°C for 5 min, 27 cycles at 95°C for 30 s, 55°C for 30 s, and 72°C for 45 s, and a final extension at 72°C for 5 min) using the primer pair 341-F 5′-CCTAYGGGRBGCASCAG-3′ and 806-R 5′-GGACTACNNGGGTATCTAAT-3′, containing an eight-base sequence barcode unique to each sample. PCR was performed in triplicate 20-μl mixtures containing 4 μl of 5X FastPfu Buffer, 2 μl of 2.5 mM dNTPs, 0.8 μl of each primer (5 μM), 0.4 μl of FastPfu polymerase, and 10 ng of template DNA. Amplicons were extracted from 2% agarose gels and purified using an AxyPrep DNA Gel Extraction Kit (Axygen Biosciences, Union City, CA, United States) according to the manufacturer’s instructions and were quantified using QuantiFluor-ST (Promega Corporation, Madison, WI, United States). Purified amplicons were pooled in equimolar amounts and paired-end sequenced (2 × 300) using an Illumina MiSeq platform according to standard protocols. Details regarding *16S rRNA* amplification, sequencing, and microbiota data analysis have been reported previously (Rühlemann et al., 2016). We obtained 8,322,002 raw reads. All raw sequencing data have been uploaded to the NCBI and SRA databases in the form of the BioProjects PRJNA515568 (16S).

### Statistical Analysis

We performed microbiome analyses using R (3.5.1.) software. Phenotypic data were expressed as the mean ± SEM using the R packages ggpubr and ggplot2. We used a pairwise *t*-test to determine significant changes between days 0 and 35 among groups. Bray–Curtis dissimilarity was calculated at the operational taxonomic unit (OTU) level using the “vegdist” function of the “vegan” package in R. Permutational multivariate ANOVA (PERMANOVA) was used to detect differences in beta diversity between groups using the “adonis” function and 10,000 permutations. Microbe composition was assessed using principal coordinate analysis using the “dudi.pco” function. A generalized linear model (GLM) was used to determine the global main and interactive effects of cold and exercise on microbe α-diversity using the “glm” function. Identification of differentially abundant phyla between room temperate and cold exposure stratified by exercise was performed using a generalized estimating equation (GEE) model with the “geeglm” function. Analysis of cold exposure versus room temperature group samples with or without exercise based on abundance changes between the end (day 35) and beginning (day 0) of the intervention was performed using the “wilcox.test” function. For Spearman correlation analysis, we used the cor.test function in base R software. In box plots, the central lines represent medians, the limits are the first and third quartiles [interquartile range (IQR)], whiskers represent the lowest and highest values within a 1.5 × IQR range ± the first/third quartile, and points are values outside the 1.5 × IQR range ± the first/third quartile.

## Results

### Effects of Cold Exercise on the Weight Loss, Cardiovascular Health, and Beige Fat Formation of Obese Rats

**FIGURE 1:**
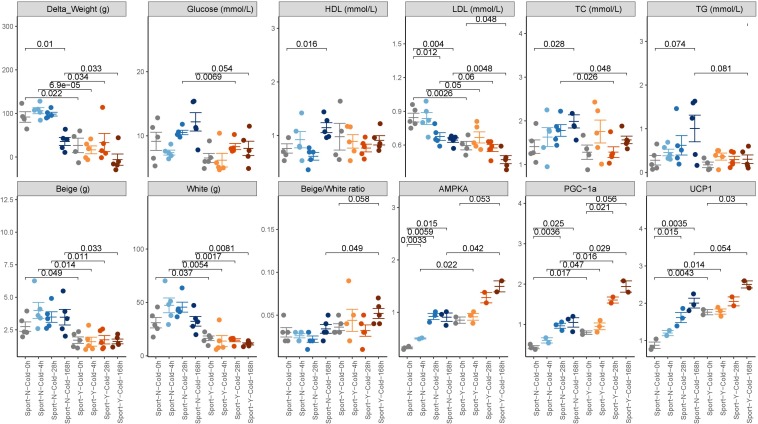
Phenotypic changes in response to cold exercise. The phenotypic data are expressed as the mean ± SEM, as determined using a two-tailed Mann–Whitney test.

Obese rats that had been fed a high-fat diet for 12 weeks were randomly assigned to eight different groups (five rats per group) and subjected to different combinations of degree of cold exposure and exercise. We collected the clinical index, blood, fat, and fecal samples before and after intervention. We accordingly found that whereas sustained cold exposure alone led to a significant decrease in weight (*P* < 0.01), intermittent or acute cold had no significant effects. Furthermore, we found that exercise reduced weight at both room (*P* = 0.022) and cold temperatures (*P* = 0.000069 for acute cold, 0.034 for intermittent cold, and 0.033 for sustained cold) (Figure 1). Moreover, a combination of cold exposure and exercise was observed to promote an increase in energy expenditure compared with either treatment alone.

Both cold exposure and exercise can individually promote the formation of beige fat, thereby burning stored fat and likely protecting mammals from hypothermia, obesity, and metabolic complications (Chevalier et al., 2015). In the present study, we found that the beige/white ratio was slightly higher in rats in the cold + exercise group than in the room temperature + exercise group (*P* = 0.058; Figure 1), although no significant differences were detected with respect to beige and white fat *per se*. Irrespective of the temperature to which the rats were exposed, exercise promoted a reduction in both beige and white fat (*P* < 0.05; Figure 1), whereas an increase in the beige/white ratio was detected only in cold-exposed rats (*P* = 0.049; Figure 1). Both cold and exercise induced BAT and inguinal fat, as well as the expression of UCP1, AMPK, and PGC-1α proteins in mice (*P* < 0.05; Figure 1), which indicates that cold and exercise promote the formation of beige fat (Fernandez-Marcos and Auwerx, 2011; Van Dam et al., 2015).

We also detected increases and decreases in the cardiovascular risk factors high-density lipoprotein (HDL) and low-density lipoprotein (LDL) cholesterol, respectively, in response to both exercise and cold exposure (*P* < 0.05; Figure 1). However, increases in total cholesterol (TC) and triglyceride levels were only observed in response to cold exposure (*P* = 0.028 and 0.074, respectively). Exercise performed in conjunction with cold exposure was observed to protect against the negative cardiovascular effects of cold exposure (*P* = 0.048 for TC, 0.081 for TG). Although we found no evidence of a modulatory effect of cold on blood glucose, enhanced glucose was observed in response to exercise (*P* = 0.054), which is deemed to be beneficial for cardiometabolic health.

We also found that exercise performed in conjunction with cold exposure promoted a greater weight loss compared with cold exposure (*P* = 0.033) or exercise (*P* = 0.17) alone and was also associated with a greater increase in the beige/white ratio and the expression of UCP1, AMPK, and PGC-1α proteins and a greater decrease in LDL cholesterol levels compared with either cold (*P* < 0.05) or exercise (*P* < 0.05) alone.

### Cold Exercise Alters the Gut Microbiome

Fecal samples were collected from the 40 experimental rats at baseline (day 0), intervention days 1 and 3, and at the end of the study (day 35) (a total 160 samples). To investigate the effects of exercise and cold temperature on the rat gut microbiota, we amplified variable regions 3–4 (V3–V4) of the bacterial *16S rRNA* gene and sequenced the products using the Illumina MiSeq platform. From a total of 8,322,002 raw reads, we observed 24,366 distinct OTUs.

**FIGURE 2:**
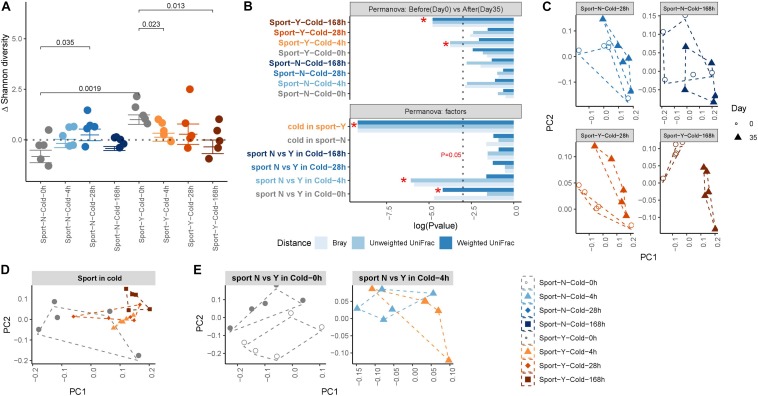
Composition of the gut microbiota shaped by cold exercise. **(A)** Changes in Shannon diversity, defined as the end point Shannon index value minus the first day Shannon index value. **(B)** Permutational multivariate ANOVA (PERMANOVA) of gut microbe beta-diversity between intervention and baseline within and between groups. The dotted line represents *P < 0.05. Label colors represent the different experimental groups. **(C–E)**. Principal coordinate analysis of unweighted UniFrac revealed clustering of the gut microbiota after cold exercise. Each dot represents a fecal community.

We subsequently performed principal coordinate analysis of unweighted and weighted UniFrac and Bray–Curtis distances between samples from the different groups or within the same group to determine the effects of exercise and cold on the microbiota. PERMANOVA was performed to determine differences in microbiota structure among the different groups. We accordingly found that both exercise (*P* = 0.0083) and cold (*P* = 0.0622) promoted marked post-intervention shifts in gut microbiota composition (Figure 2C), and that cold exercise (adonis *R* = 0.285) was associated with considerably more extensive attenuation of alterations in the microbiota than either cold (adonis *R* = 0.148) or exercise (adonis *R* = 0.17) alone (Figure 2B and Supplementary Table S1). At the end of the intervention, we detected a clear separation between the communities in response to cold (*P* = 0.0001) and exercise treatments, but not in the no-exercise groups (Figures 2B,D). Exercise was found to modify the composition of gut microbiota at room temperature (*R* = 0.136, *P* = 0.0091; Figure 2E), whereas when performed in conjunction with cold exposure, cold reduced the major alterations observed at room temperature (*R* = 0.052, *P* = 0.2114), thereby indicating the interactive effect of cold and exercise with respect to the modification of gut microbiota composition.

### Exercise Reversed the Alterations in Gut Microbiota Induced by Cold Exposure

To further investigate the effects of exercise and cold exposure on the phylogenetic richness of the gut microbiota, we performed α-diversity analyses, by assessing the changes in Shannon diversity that occurred as a consequence of an intervention (Figure 2A). We accordingly observed a marked increase in microbial α-diversity in response to exercise at room temperature (*P* = 0.0019) but not when performed in conjunction with cold exposure. Intermittent cold alone was found to increase microbial α-diversity (*P* = 0.035), whereas sustained cold combined with exercise resulted in a marked decrease in α-diversity (*P* = 0.013). To assess the global main and interactive effects of cold and exercise on microbe α-diversity, we used a GLM (see Supplementary Table S2) and accordingly found that cold exposure increased α-diversity independently of exercise (*P* = 0.049) and that exercise increased α-diversity independently of cold (*P* = 0.0001). However, the interactive effect of cold and exercise indicated that exercise reversed the increase in α-diversity of the gut microbiota promoted by cold exposure (*P* = 0.00149).

**FIGURE 3:**
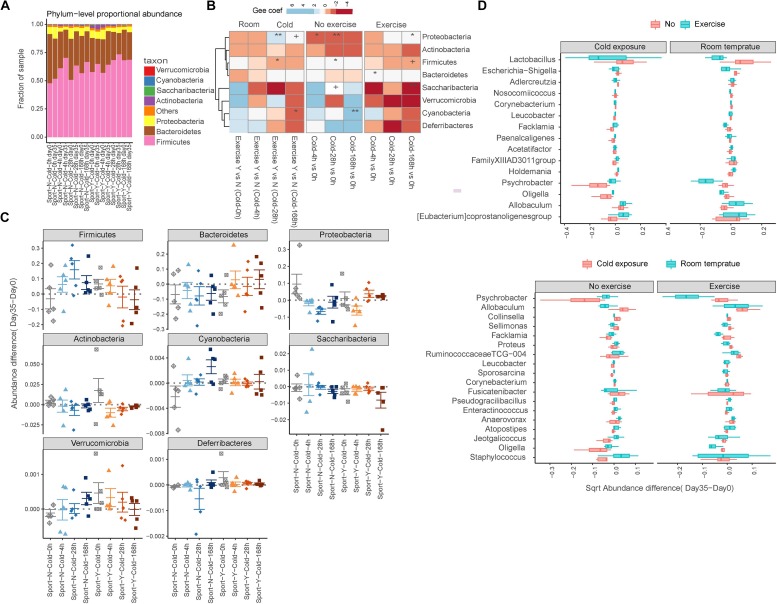
Exercise reversed the alterations in gut microbiota in response to cold exposure. **(A)** Relative abundance at the phylum level. **(B)** Comparison of phylum-level proportional abundance in feces up to 35 days in response to cold exercise, cold exposure alone, exercise alone, or in control rats. The blue color denotes an increase, and the red color denotes a decrease. +*P* < 0.1; *P < 0.05; **P < 0.01. **(C)** Richness is represented as the proportion of operational taxonomic units (OTUs) classified at the phylum level. **(D)** Box plots showing the transformed relative abundance change in representative genera following intervention selected for the first 15 most important genera.

Analysis at the phylum level indicated that the gut microbiota of rats was dominated by four major phyla, namely, *Firmicutes*, *Bacteroidetes*, *Proteobacteria*, and *Actinobacteria* (Figure 3A). Similar to the observed changes in α-diversity, we found that exercise reversed the shifts in seven of eight phyla upon cold exposure. On the basis of a GEE model, we found that whereas increases in the abundances of *Verrucomicrobia*, *Deferribacteres*, and *Cyanobacteria* were detected in response to either cold exposure or exercise alone (Munukka et al., 2018), all these decreased in response to cold exercise (Chevalier et al., 2015; Ziętak et al., 2016; Worthmann et al., 2017; Figures 3B,C). Furthermore, the abundances of *Firmicutes* and *Saccharibacteria* increased in response to cold exposure (*P* = 0.041 and 0.081, respectively) but decreased in response to cold exercise (*P* = 0.094 and 0.008, respectively) (Chevalier et al., 2015; Ziętak et al., 2016; Supplementary Table S3). In contrast, however, *Proteobacteria* decreased in response to cold exposure alone (*P* = 0.0002) but increased in response to cold exercise (*P* = 0.040) (Chevalier et al., 2015; Ziętak et al., 2016).

Among the 178 microbial genera detected, we observed significant differences in 38 genera, as determined using the Wilcoxon rank sum test, performed across the cold exposure versus room temperature group samples with or without exercise based on the changes in abundance between the start and end of intervention (*P* < 0.05; Supplementary Table S4). Among these genera, we observed that shifts in the direction of *Psychrobacter*, *Facklamia*, *Proteus*, *Ruminococcaceae TCG-004*, *Jeotgalicoccus*, and *Oligella* in response to cold exposure were reversed by exercise (Figure 3D). Furthermore, the abundance of the genera *Allobaculum*, *Collinsella*, *Sellimonas*, and *Fusicatenibacter* increased, whereas that of *Enteractinococcus*, *Atopostipes*, and *Staphylococcus* decreased in response to cold exposure independent of exercise. Comparably, the abundances of *Lactobacillus* and *Escherichia–Shigella* decreased and those of *Holdemania*, *Allobaculum*, and *Eubacterium coprostanoli* increased in response to exercise independent of cold exposure.

### Correlations Between Cold Exercise-Related Changes in Gut Microbial Communities and Clinical Phenotypes

**FIGURE 4:**
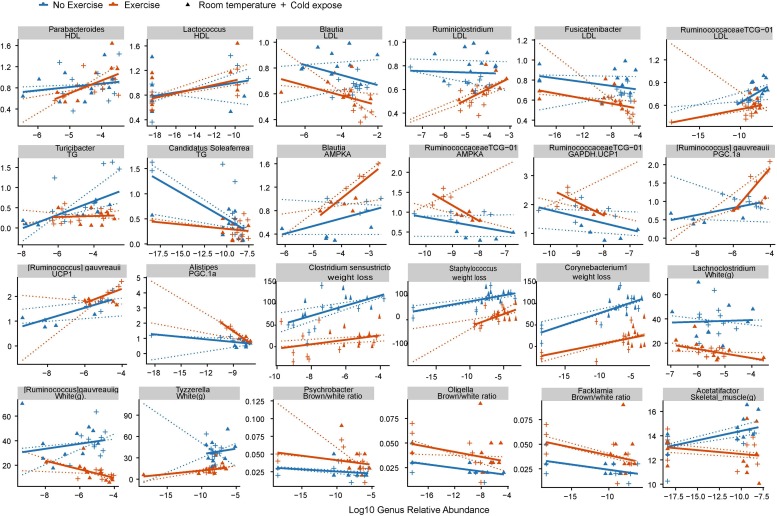
Correlations between cold exercise-related changes in gut microbial communities and clinical phenotypes. Spearman correlation was used to analyze the association between the cold exercise-induced changes in bacterial genera and phenotypes. Cold exercise-induced genera were identified based on a *q* < 0.1 value using the Wilcoxon rank sum test between groups stratified according to temperature or exercise. Each point represents a fecal sample. Solid lines indicate a robust linear regression within two groups with or without exercise. Dotted lines are robust linear model (RLM) within the control, cold exposed, exercise, cold exercise groups. The *X*-axis denotes the relative abundance of genera, and the *Y*-axis denotes phenotypes.

Using Spearman correlation analysis, we subsequently examined the correlations between cold exercise-related changes in the abundance of bacterial genera (*P* < 0.1; Supplementary Table S4), weight loss, cardiovascular risk, and formation of beige fat collected at the end of the study (day 35) (*P* < 0.01; see Supplementary Table S5). We accordingly found that 25 of the 68 significant correlations were reversed in groups with or without exercise, which indicated an exercise-specific association with microbial health. Abundances of the exercise-enriched genus *Parabacteroides* and the cold-enriched genus *Lactococcus* were positively correlated with HDL cholesterol levels, which are deemed beneficial to cardiovascular health (Supplementary Table S5 and Figure 4). The cold exercise-enriched genus *Blautia* and cold-enriched *Fusicatenibacter* were shown to be negatively correlated with LDL cholesterol levels, which are considered detrimental to cardiovascular health. Cold exercise also promoted a decrease in *Ruminococcaceae TCG-013*, which was positively correlated with LDL cholesterol, whereas the genera *Candidatus* and *Soleaferrea*, the abundances of which were also promoted by cold exercise, were negatively correlated with total triglyceride levels, which are considered detrimental to cardiovascular health. Collectively, these observations thus indicate that the changes in microbial abundance attributable to cold exercise have a positive effect on cardiovascular factors.

The cold exercise-enriched genus *Blautia* and cold-enriched species *Ruminococcus gauvreauii* were also shown to be positively correlated with expression levels of the white fat browning proteins AMPKA and UCP1. Furthermore, we found that *Ruminococcaceae TCG-013*, the abundance of which decreased in response to cold exercise, was negatively correlated with AMPKA, UCP1, and glucose, whereas the cold-enriched *Clostridium sensustricto*, *Staphylococcus*, and *Corynebacterium* were found to be positively correlated with weight loss. Unexpectedly, we found that decreases and increases in the genera *Psychrobacter*, *Oligella*, and *Facklamia* in response to cold exposure and exercise, respectively, were negatively correlated with beige/white fat ratio.

## Discussion

Although previous studies have demonstrated that changes in the gut microbiota in response to cold exposure can contribute to reducing body weight (Chevalier et al., 2015; Ziętak et al., 2016; Worthmann et al., 2017) and that exercise-induced changes in microbiota have favorable cardiovascular effects (Taniguchi et al., 2018), there is currently little information available regarding the combined effects of cold exposure and exercise on the gut microbiota or the associations between microbial populations modified in response to cold exercise and systemic metabolism. Thus, characterization of the changes in the gut microbiota in response to cold exercise and the following clinical outcomes is highly warranted.

Previous studies have shown that both acute cold exposure and intermittent cold exposure have a significant impact on fat oxidation or fat accumulation (Ravussin et al., 2014; Yoo et al., 2014; Acosta et al., 2018). Furthermore, exercise performance has been found to be influenced by acute and chronic cold exposure (Wakabayashi et al., 2015). Here, we followed a study design similar to that of published studies. Diet-induced obese rats were observed consecutively for room temperature, acute cold, intermittent cold, sustained cold, combined with or without exercise, resulting in a total of eight subgroups of data on white fat browning and weight loss at four time points during experimental trials (days 0, 1, 3, and 35), which we believe is sufficient to represent a robust account on fat treatment. One of the limitations of the study is the sample size in each group, which was almost five rats per group. However, we believe that the longitudinal monitoring (four time points for each rat, 4 × 5 = 20 samples per group) of protein expression data, weight measurement, and sampling of gut microbiota extended our insights beyond what has been reported so far, where most studies lacked gut microbiota data and cross-sectional designs. The high-fat diet was controlled and was almost the same for each group of rats (23.436 ± 4.109 g).

In this study, we found cold exercise to be more beneficial than either cold exposure or exercise alone in terms of promoting weight loss, beige fat formation, and blood lipid markers indicating cardiovascular health, whereas, consistent with previous observations (Kaisanlahti and Glumoff, 2019), we found that cold exposure may have detrimental cardiovascular effects *via* the promotion of higher levels of cholesterol and triglyceride, which are depleted in response to exercise. Unexpectedly, we found that cold exercise promoted shifts in the gut microbiome that were considerably more extensive than those observed in response to exercise or cold exposure alone. Moreover, we detected considerable reverse shifts in the α-diversity of major bacterial phyla, as well as certain genera, in response to cold exercise. Cold exposure promotes an increase in fecal bile acid excretion and shapes the gut microbiome (Worthmann et al., 2017), and exercise produces serum lactate, which transverses the epithelial barrier into the lumen of the gut and can thus be metabolized by certain strains of gut bacteria (Scheiman et al., 2019). However, the active major pathways stimulated in response to exercise performed in conjunction with cold exposure have yet to be determined. Nevertheless, since the associated increases in bile acid and lactate excretion or the activation of alternative undetected metabolic pathways are not known, it is difficult to unravel the effects of cold exercise with respect to the observed extensive shifts in gut microbiome composition. Given that the changes in gut microbial abundance in response to cold exercise were also found to be associated with weight loss, beige fat formation, and cardiovascular health, our observations suggest the possible operation of metabolic feedback processes related to the cold exercise-induced changes in the abundance of certain bacterial strains. In this regard, *Veillonella* atypical, the abundance of which increases in response to exercise, has been demonstrated to enhance running time performance *via* its metabolism of lactate (Scheiman et al., 2019), whereas *Akkermansia muciniphila*, which is reduced in abundance in response to cold exposure, is known to be associated with intestinal gene expression promoting tissue remodeling and suppression of apoptosis *via* a currently unknown pathway (Chevalier et al., 2015). Besides, other components such as lipopolysaccharide of diet-induced obesity-related gut bacteria may promote inflammation (Boulangé et al., 2016), However, the obesity-related inflammation is reversed by exercise (Kawanishi et al., 2012), ketogenic diet (Ma et al., 2018), or food components such as green tea polyphenols (Hung et al., 2018), which might be due to certain modulations by the gut microbiome. Microbiota depletion has been shown to promote the browning of white adipose tissue and to reduce obesity (Suárez-Zamorano et al., 2015). One study determined the gut microbiome fermentation products acetate and lactate and the selective upregulation of monocarboxylate transporter 1 expression in beige cells (Li et al., 2017). We also noticed the improvement of blood lipid markers of cardiovascular health in the cold exercise group and correlation between this improvement and some bacteria, which might be due to the conversion of cholesterol to fecal bile acid excretion, thereby changing the gut bacterial composition (Worthmann et al., 2017). Collectively, these observations indicate that the superior health effects linked to cold exercise may depend on extensive shifts in gut microbe populations and a related enhancement of the interactions among certain microbial species. Further studies are clearly warranted to delineate how cold exercise promotes such a modulation of microbial metabolism.

Although many questions remain unanswered, we believe that the findings of this study make a valuable contribution to advancing our current knowledge on the inter-relationships among cold exercise, gut microbiota, and systemic metabolism and provide a basis for developing possible future modalities for weight loss intervention by targeting specific components of the gut microbiota.

## Data Availability Statement

The datasets generated for this study can be found in the PRJNA515568.

## Ethics Statement

This study was carried out in accordance with the principles of the Basel Declaration and recommendations of Guangzhou Sport University protocol. The protocol was approved by the Guangzhou Sport University ethics committee.

## Author Contributions

XW contributed to the study design. YM, LC, WL, HW, and GX contributed to the data collection. XW and YM performed the statistical analyses. All authors provided input on the manuscript and have reviewed and approved the final draft.

## Conflict of Interest

HW was employed by the company Shenzhen Health Time Gene Technology Co., Ltd. The remaining authors declare that the research was conducted in the absence of any commercial or financial relationships that could be construed as a potential conflict of interest.
